# Hybrid AI Model With CNNs and Vision Transformers for Precision Pest Classification in Crops

**DOI:** 10.1002/fsn3.71174

**Published:** 2025-11-09

**Authors:** Neha Sharma, Fuad Ali Mohammed Al‐Yarimi, Salil Bharany, Ateeq Ur Rehman, Belayneh Matebie Taye

**Affiliations:** ^1^ Chitkara University Institute of Engineering and Technology Chitkara University Punjab India; ^2^ Applied College of Mahail Aseer King Khalid University Muhayil Aseer Saudi Arabia; ^3^ School of Computing Gachon University Seongnam‐si Republic of Korea; ^4^ Department of Computer Science, College of Informatics University of Gondar Gondar Ethiopia

**Keywords:** attention mechanism, channel attention, convolutional neural network, crop pest, spatial attention, vision transformer

## Abstract

Crop pests pose a significant threat to agricultural productivity, making it essential to develop effective pest management techniques. Prompt and accurate identification of pests is necessary for effective pest management and preventing significant damage. This paper proposes HyPest‐Net, a hybrid deep learning architecture that integrates convolutional neural networks (CNNs) for local feature extraction, channel and spatial attention mechanisms for refining salient features, and a vision transformer (ViT‐B/16) module for modeling long‐range dependencies. This integrated hybrid architecture enables accurate pest classification by resolving challenges posed by visually similar species, background clutter, and varied illumination issues that standalone CNNs or ViTs inadequately address. Preprocessing and augmentation have been used to enhance the generalizability of the proposed model over the dataset. The proposed model was evaluated on two benchmark datasets: a rice pest dataset (5 classes) and the dangerous farm insects dataset (15 classes). Experimental results demonstrate that HyPest‐Net achieved an accuracy of 0.95 on the rice pest dataset. The proposed model achieved a precision of 0.95, a sensitivity of 0.95, a specificity of 0.94, and an F1 score of 0.94. The proposed model achieved an accuracy of 0.93 on the dangerous farm insects dataset. The proposed HyPest‐Net model offers a lightweight yet powerful solution for real‐time, explainable pest classification, supporting practical applications in precision agriculture.

## Graphical abstract

This study proposes a hybrid deep learning model integrating CNNs, attention mechanisms, and Vision Transformers for accurate classification of five major rice crop pests. The model captures both fine‐grained local features and global spatial relationships, achieving a high accuracy of 95% on an augmented dataset. Results demonstrate its effectiveness over traditional CNNs, offering a scalable and robust solution for precision pest management in agriculture.

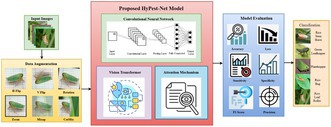

## 1 Introduction

Agriculture plays a crucial role in sustaining the global economy by ensuring food security, generating employment, and promoting economic stability. It serves as the backbone of many countries, particularly in developing economies, where the majority of the population depends on farming for survival. Despite its significance, the agricultural sector continues to face persistent challenges, including land degradation, water scarcity, climate change, and biological threats, such as crop pests and diseases. Among these, crop pests are one of the most damaging biological stressors, responsible for billions of dollars in annual yield losses worldwide (Mallick et al. 2023; Rimal et al. 2023). They disrupt food value chains and the livelihoods of millions of smallholder farmers, and their increasing frequency due to climate change necessitates timely and effective pest control measures. Conventional methods for identifying pests rely heavily on manual processes based on expert eye and field inspection. Although these have been core, they are time‐consuming, labor‐intensive, subjective, and do not deliver real‐time information.

Additionally, the traditional reliance on chemical pesticides has led to severe issues, including soil pollution, water contamination, pesticide resistance, and harm to nontarget species, such as beneficial pollinators (Nanni et al. 2020; Liu, Wu, et al. 2020). These drawbacks indicate a need for intelligent and more scalable alternatives. In response, recent developments in the field of artificial intelligence have introduced the prospect of automated pest recognition systems, which have the potential to revolutionize pest management into an action‐oriented and effective process. In the past decade, deep learning has emerged as a dominant paradigm in complex visual recognition tasks across multiple domains, including agriculture. Among these, convolutional neural networks (CNNs) have shown exceptional performance in image‐based tasks such as disease diagnosis, crop classification, and yield estimation (Kamilaris and Prenafeta‐Boldú 2018; Li et al. 2019; Liu, Xie, et al. 2020). CNNs hierarchically extract spatial features, such as textures, edges, and contours, using layered convolutional and pooling operations.

These networks perform particularly well in learning high‐fidelity local features—such as wing venation, color patches, and body structures—that are also essential for pest classification. CNNs, however, are intrinsically limited by their receptive field and are therefore less adept at modeling global spatial relationships within images. To address this problem, the community has increasingly turned to vision transformers (ViTs), which represent a paradigm shift from convolutional to attention‐based models. ViTs split images into fixed‐sized patches and compute them with multi‐head self‐attention mechanisms. Contrary to CNNs, ViTs can perceive long‐range dependencies and global context in the image. This is especially useful in pest classification problems, where a large number of pests can have similar local features but can be distinguished based on their overall posture, body alignment, or spatial organization. To complement ViTs, attention mechanisms in the form of channel attention and spatial attention have also been proposed to further enhance CNN performance (Nieuwenhuizen et al. 2018).

Channel attention targets highlight key feature channels, such as color strength or texture intensity, whereas spatial attention accentuates notable regions within the image, such as the wings, legs, and antennae of a pest. This enhances the model's ability to disregard irrelevant background features and focus on the biologically relevant portions of the pest, thereby improving interpretability and classification performance in field settings. Earlier studies in pest classification have used isolated CNNs, handcrafted feature‐based machine learning models (e.g., SVMs, decision trees), and transfer learning. Although each of these has proved to be useful, they suffer from disadvantages such as poor generalization, high computational expense, and a lack of representation of global dependencies (Liu et al. 2019). Certain works have attempted to enhance accuracy through ensemble methods or feature combination, but these tend to sacrifice efficiency and interpretability (Li et al. 2022). Therefore, the use of hybrid models that integrate CNNs with attention mechanisms and ViTs is gaining prominence as a viable approach to achieve high accuracy while maintaining scalability (Pacal and Işık 2025; Pacal et al. 2024). Herein, we introduce an unsupervised hybrid deep learning model that integrates a self‐crafted CNN, channel and spatial attention, and a vision transformer (ViT‐B/16) to predict crop pests with high accuracy. The CNN module is used to extract localized features, such as body textures, wing form, and color gradients. Attention mechanisms reinforce the representations by weighing pest‐relevant features higher and downplaying noisy background effects. Lastly, the ViT module combines these features from the whole image space to obtain global contextual information, leading to strong classification even for morphologically similar pests. The model is trained and tested on a custom‐curated dataset of five economically harmful rice pest species: rice stem borer, green leafhopper, planthopper, rice bug, and rice leaf roller.

Every pest has a specific challenge related to visual identification and field recognition. The rice stem borer, for instance, produces “dead heart” symptoms through the destruction of the plant's vascular system, causing yield loss of up to 80%. Tungro virus is transmitted by the green leafhopper, causing stunted growth. Planthoppers cause “hopper burn” and can devastate entire fields under heavy infestation. Rice bugs feed on developing grains, reducing market value due to discoloration, while rice leaf rollers impair photosynthesis by rolling and feeding within leaves. These pests contribute collectively to substantial crop damage, reduced grain quality, and financial losses in rice‐producing economies. Timely and accurate identification of these pests is therefore critical. Our model addresses this by providing a real‐time, automated, and accurate classification system that can operate in complex environmental conditions. By eliminating dependency on manual inspection, the proposed system facilitates early intervention, optimizes pesticide application, and improves resource efficiency.

In summary, the novelty of our approach lies in the strategic integration of CNNs, attention mechanisms, and ViTs, which combine the strengths of local feature extraction, spatial saliency enhancement, and global context modeling. The model is not only highly accurate and interpretable, but also scalable and suitable for deployment in real‐world agricultural systems such as drone‐based monitoring, smart farming platforms, or edge AI devices. It provides an effective and sustainable solution for precision pest management, contributing directly to economic resilience and agricultural sustainability.

This paper is structured as follows: the subsequent section reviews pertinent literature about insect detection utilizing deep learning models. Section 3 delineates the methods employed in formulating the proposed model, encompassing data preparation, model design, and training protocols. Section 4 presents the experimental findings and analyzes the efficacy of the proposed paradigm. Section 5 concludes the work by outlining prospective avenues for further research in this domain.

## 2 Literature Review

The task of categorizing crop pests using deep learning techniques has been the focus of research over the last few years, with many researchers making efforts to improve the accuracy, stability, and efficiency of pest classification algorithms (Kunduracioglu and Pacal 2024; Pacal 2024). The need for computer vision‐based automated pest recognition has led to the evolution of CNNs, attention models, and transformer models, each conferring different advantages. This section provides an overview of some significant advances in this field, highlighting CNN‐based approaches, hybrid deep models, and advanced methodologies that combine transfer learning, attention mechanisms, and ensemble learning approaches for improved pest classification.

One of the first deep learning models for pest categorization was presented by Thenmozhi who used three open‐source datasets: NBAIR (40 classes), Xie1 (24 classes), and Xie2 (40 classes) to develop a CNN‐based classifier for insects (Thenmozhi and Reddy 2019). The model utilized data augmentation to enhance generalization and prevent overfitting, resulting in improved classification accuracy compared to pretrained models such as AlexNet, ResNet, GoogLeNet, and VGGNet. Transfer learning was highlighted by the authors, where pretrained networks boosted classification significantly when fine‐tuned for pest detection. In another thread of research, Xie et al. (2018) investigated unsupervised feature learning for pest classification, where sparse coding represented low‐level pest image features, which were then incorporated into a multilevel classification framework. They proposed a multi‐scale alignment‐pooling mechanism in their work, which rectified patch‐level feature misalignments and resulted in enhanced classification of 40 crop pest species. According to the study, misalignment in patch‐based classification models may contribute to a reduction in accuracy, while using alignment‐pooling techniques significantly improves classification performance. Likewise, Malek et al. (2021) designed a pest classification system using CNN that was trained on 9500 images to classify 20 species of pests as either harmful or beneficial. The model achieved 90% accuracy, which was higher than traditional pest detection measures. The article highlighted the importance of utilizing large datasets and noted that deep learning models require sufficient training with ample data to generalize effectively to previously unseen pest species. To further enhance classification accuracy, researchers have examined hybrid deep learning models integrating CNNs with multi‐scale feature extraction mechanisms. Wei et al. (2022) introduced the multi‐scale feature fusion network (MFFNet), which employs dilated convolutions to capture multi‐scale features and fuses them with deep feature maps, yielding improved pest classification accuracy. Their technique achieved 98.2% accuracy on a dataset of 12 classes of pests, demonstrating the potential for employing multi‐scale feature extraction techniques to detect pests regardless of environmental conditions. Ullah et al. (2022) developed a hybrid algorithm with the creation of DeepPestNet, an 11‐layer deep CNN architecture, which was also trained and tested on Deng's crop dataset (containing 10 pest species) (Ullah et al. 2022). It was trained on data augmentation methods, such as rotation and scaling, to enhance generalizability, and achieved 100% classification accuracy. The work also identified the role played by deep architectures and data augmentation methods in enabling the training of pest classification models that can deal with real‐world, diversified situations.

Transfer learning has been explored in several studies, where pretrained deep models are adapted for pest classification. Seven pretrained CNN models (VGG‐16, VGG‐19, ResNet‐50, Inception‐V3, Xception, MobileNet, and SqueezeNet) were also compared on the D0 dataset (40 pest categories) (Ayan et al. 2020). They achieved 98.81% accuracy through ensemble learning, combining probability summation and a genetic algorithm, and demonstrated that ensemble learning can significantly enhance pest classification. The technique was also tested on a small dataset (10 classes) and the IP102 dataset (102 classes), achieving accuracies of 95.15% and 67.13%, respectively. The research demonstrated that pretrained models, when properly combined, can outperform single deep learning architectures. Likewise, authors proposed Faster‐PestNet, an improved Faster R‐CNN model with MobileNet as its backbone, and achieved an accuracy of 82.43% on the IP102 dataset (Ali et al. 2023). The model proved robust against size differences, lighting conditions, and image distortions, demonstrating the value of employing lightweight, efficient pretrained networks for pest classification. State‐of‐the‐art developments in deep learning have led to the use of attention mechanisms in CNN‐based structures. Rani et al. presented U‐SegNet, a compound U‐Net and ResNet‐based model incorporating hyperparameter adjustment and data expansion to enhance the accuracy of pest classification (Rani et al. 2023). Their model was 93.54% accurate, specifically targeting misclassification problems caused by morphological similarity among pests. The authors emphasized the importance of accurate segmentation in pest classification, particularly in precision agriculture use cases, where incorrect pest identification would lead to false pesticide application. Another segmentation‐based research study by Kasinathan et al. (Kasinathan et al. 2021) proposed an insect pest detection method that utilizes foreground extraction and contour detection to identify pests in cluttered agricultural backgrounds. The model achieved 91.5% classification accuracy for nine classes of insects and 90% accuracy for 24 classes of insects using 9‐fold cross‐validation. The authors found that background clutter in actual crop images can significantly impact classification performance, and methods such as foreground extraction can enhance model robustness. Ensemble learning has been investigated to improve performance for pest classification. Khanramaki et al. (Khanramaki et al. 2021) proposed an ensemble classifier based on deep CNNs for citrus pest classification, with classifier‐level, feature‐level, and data‐level diversities. The work utilized a dataset of 1774 images of citrus leaves, with an accuracy of 99.04% based on 10‐fold cross‐validation. The results indicated that ensemble learning enhances classification accuracy by utilizing multiple models compared to a single deep network. Likewise, Kundur et al. (Kundur and Mallikarjuna 2022) introduced Faster R‐CNN models for the classification of pests, using EfficientNet B4 and B7, and tested them on 5‐, 10‐, and 15‐class subsets of the IP102 dataset. Their EfficientNet B7 model achieved classification accuracies of 99.00%, 96.00%, and 93.00%, respectively, surpassing those of conventional CNN architectures. The research demonstrated the effectiveness of EfficientNet‐based object detection models in agricultural and pest classification tasks.

Although deep learning has been widely used for crop pest classification, current models primarily rely on CNN‐based architectures or transfer learning methods that are limited in handling background noise, morphological resemblance among pest species, and generalization to novel data. Some studies have investigated CNNs combined with conventional feature extraction techniques; however, they are incapable of capturing long‐range dependencies in pest images, which hinders their ability to separate visually similar species. Other research has utilized ensemble methods and pretrained networks to improve classification accuracy. However, they do not completely solve the problem of feature refinement and robustness to varied environmental conditions. The proposed model distinguishes itself from prior research by combining CNNs, attention mechanisms, and ViTs to form a hybrid architecture that efficiently balances the extraction of local and global features.

In contrast to traditional CNNs, which are based on localized receptive fields, our model enhances feature selection through channel and spatial attention mechanisms, allowing the model to emphasize the most relevant pest features and filter out background noise. Furthermore, the integration of ViTs enables global self‐attention processing to capture structural relationships and long‐range dependencies throughout an entire image, which is particularly useful for pest species with similar local textures but dissimilar overall morphologies. Another major differentiating factor is our data augmentation policy, which provides model generalizability by injecting variability in lighting, orientation, and background conditions to counter dataset bias and enhance real‐world applicability. A detailed comparison of recent pest classification studies, including model types, datasets, performance metrics, and research limitations, is presented in Table 1. In addition, this research contributes to scalable pest classification systems by developing an extensible framework that can be applied in real‐time use, IoT‐based monitoring, and edge computing solutions. Besides outperforming conventional CNN‐based methods in terms of classification accuracy, this hybrid deep learning method opens up the door for smart, autonomous pest monitoring systems that can be flexible across various agricultural settings and changing pest trends.

**TABLE 1:** Literature review.

Ref/Year	Technique/Model	Dataset used	Accuracy	Research gap addressed
Thenmozhi and Reddy (2019)/2020	CNN vs. AlexNet, ResNet, VGG	NBAIR, Xie1, Xie2 (24–40 Classes)	97.47%	No attention mechanisms or global feature modeling
Xie et al. (2018)/2018	Sparse Coding+ Multi‐scale Alignment	Custom (40 pest species)	—	Lacks deep learning; patch‐level misalignment.
Malek et al. (2021)/2021	Custom CNN	9500 images (20 pests)	90%	No use of attention or transformer models
Wei et al. (2022)/2022	MFFNet (Multi‐scale feature fusion)	12 pest species	98.2%	No attention modules
Ullah et al. (2022)/2020	DeepPestNet (11‐layer CNN)	Deng's crop dataset (10 pests)	100%	Dataset‐specific; lacks generalization tools
Ayan et al. (2020)/2022	Ensemble of 7 CNNs + GA	D0, SMALL, IP102	98.81%	High computation cost; lacks interpretability
Ali et al. (2023)/2023	Faster R‐CNN + MobileNet	IP102	82.43%	Lower accuracy on multiclass datasets
Rani et al. (2023)/2021	U‐SegNet (U‐Net + ResNet)	Custom insect dataset	93.54%	Focuses on segmentation
Kasinathan et al. (2021)/2021	Contour‐based pest detection	24 insect classes	91.5%	Sensitive to background clutter
Khanramaki et al. (2021)/2021	Ensemble CNN (feature & classifier‐level)	Citrus pest dataset (1774 images)	99.04%	High complexity
Kundur and Mallikarjuna (2022)/2022	EfficientNet B4 + Faster R‐CNN	Subsets of IP102	99%	High resource demand

Based on the identified limitations in the existing literature, such as insufficient generalization, background sensitivity, and lack of global context modeling, this study presents the following key contributions:

- A hybrid deep learning model that integrates CNNs, attention mechanisms (channel and spatial), and ViTs to improve crop pest classification accuracy and robustness.
- A lightweight CNN backbone designed for small pest datasets, optimized to capture fine morphological traits such as wing venation and pigmentation patterns.
- Integration of channel and spatial attention specifically to suppress dominant background vegetation and emphasize pest body regions.
- Use of a ViT head to capture global morphology, which resolves confusions between locally similar pests (e.g., planthopper vs. rice leaf roller).

## 3 Materials and Methods

The proposed model is an integrated deep learning approach that combines the CNN, attention mechanism, and ViT. This work employs an experimental research methodology, involving the design, implementation, and empirical evaluation of a deep learning‐based model for crop pest classification. This section will represent a detailed description of the proposed model, dataset, and preprocessing.

### 3.1 Input Dataset and Preprocessing

**FIGURE 1:**
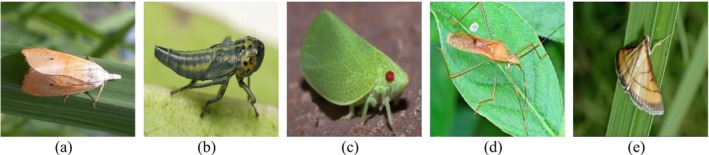
Sample images of crop pest dataset (a) Rice stem borer, (b) Green leafhopper, (c) Planthopper, (d) Rice bug, and (e) Rice leaf roller.

The crop pest dataset comprises the images used in research on identifying agricultural pests. The dataset comprises 275 images of five known pests. These pests cause serious harm to the rice crop, necessitating the importance of identifying them for prompt pest control. The dataset used in this research is a diverse set of crop pest images collected from publicly accessible datasets and field‐gathered images, ensuring real‐world applicability. Figure 1 shows some example images from a crop pest dataset, highlighting five primary pests that significantly impact agricultural yields, particularly in rice cultivation. The pests include the (a) rice stem borer, (b) green leafhopper, (c) planthopper, (d) rice bug, and (e) rice leaf roller. Each image accurately represents the pest's morphology, highlighting specific characteristics crucial for identification and classification.

The images in this dataset were obtained from the Kaggle online platform (Ghosh 2025). This dataset is designed to support the development and evaluation of machine learning models for automatic pest identification. Before inputting the images into the model, some preprocessing was performed to normalize and enhance the quality of the dataset. All images were resized to a constant resolution for uniformity of training batches, allowing the deep learning model to operate uniformly with consistent input. The original dataset was compiled from multiple sources and devices, resulting in inconsistent image resolutions ranging from 210 × 172 to 2252 × 2164 pixels. This variability in image size posed challenges for uniform input processing, batch training efficiency, and stable feature extraction across samples. To address this, we standardized all images by resizing them to a uniform size of 224 × 224 pixels. This dimension was selected to achieve a balance between preserving critical pest features (such as wing patterns, body shape, and texture) and maintaining computational efficiency during training.

Given the relatively small size of the dataset (275 images), there is a risk of both overfitting and underfitting in deep learning models. Overfitting may occur if the model memorizes training samples instead of learning generalizable patterns, while underfitting might result from insufficient learning due to limited data diversity. To address these issues, we employed several strategies: (i) extensive data augmentation techniques (random rotation, flipping, brightness, affine transformations) were applied to expand the training dataset fivefold (to 1375 images), thereby introducing variation and reducing model memorization; (ii) dropout layers and L2 regularization were incorporated in the CNN to avoid overfitting; (iii) batch normalization layers were used to stabilize training; and (iv) early stopping and adaptive learning rates were implemented during training to prevent both over‐training and underfitting. A 70:20:10 split was used for training, validation, and testing, respectively. This split was selected to ensure a balanced and statistically sound evaluation. Allocating 70% of the data for training enables the model to learn from a sufficient number of examples and capture the diversity of features across classes. The 20% validation set is used for hyperparameter tuning, early stopping, and performance monitoring, ensuring that model optimization decisions are not biased by the test set. The remaining 10% is reserved strictly for testing, providing an unbiased final assessment of the model's generalization performance. For preliminary experiments, model development, and ablation studies, the dataset was divided into training, validation, and testing subsets using a 70:20:10 split to tune hyperparameters and monitor convergence. For final performance reporting and statistical evaluation, a fivefold stratified cross‐validation protocol was employed, ensuring that each image appeared in the test set exactly once and providing a robust estimate of the model's generalization capability.

### 3.2 Data Augmentation

**FIGURE 2:**
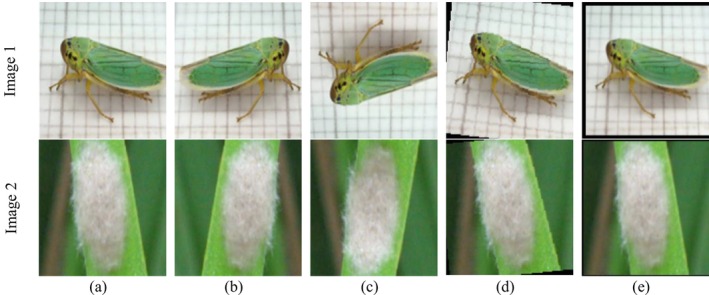
Data augmentation (a) Input images, (b) Horizontal flip, (c) Vertical flip, (d) Rotation, and (e) Zoom.

To improve the generalization capacity of the proposed model and counteract overfitting due to the small dataset size, a large data augmentation pipeline was employed. Every image was resized to 224 × 224 pixels, then random transformations were applied to it during training. The geometric augmentations involved random horizontal and vertical flippers, (±15°) rotation, and brightness. Figure 2 illustrates the geometric data augmentation process employed to enhance the diversity and robustness of the pest image dataset utilized in this research. Augmentation was used to simulate natural variations that occur in field conditions, including changes in pest orientation, viewing angle, and scale. Figure 2 shows two input samples, Images 1 and 2, along with their respective augmented versions. Figure 2a illustrates the input images to be transformed, which are the baseline samples. The applied transforms are a horizontal flip in Figure 2b and a vertical flip in Figure 2c, which produce mirror views of the bugs for the model to learn orientation‐robust representations. The rotation transformation in Figure 2d introduces angular variation (±15°) to simulate random camera viewpoints and insect body postures. In contrast, the zoom transformation in Figure 2e scales the image to simulate variations in object size and camera distance.

**FIGURE 3:**
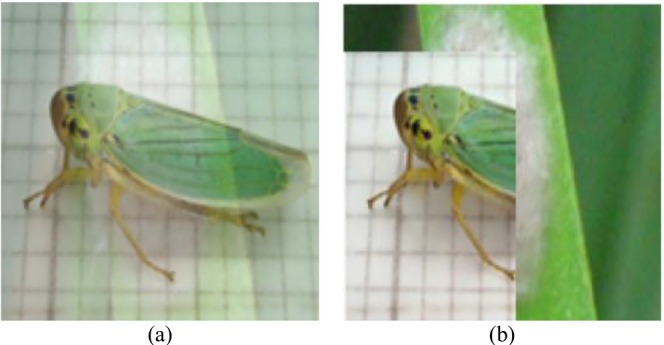
Advanced data augmentations (a) Mixup and (b) CutMix.

In addition to standard augmentation, the training procedure also included state‐of‐the‐art sample‐mixing strategies, Mixup and CutMix, to further enhance generalization and robustness. Mixup creates mixed samples by linearly interpolating pairs of images and their respective labels, which forces the model to have a smoother decision boundary and is less sensitive to noise. CutMix, however, substitutes random rectangular areas of an image with patches from a different image, essentially fusing local texture and structural cues from two samples. These hybrid data augmentation techniques significantly enhance intra‐class variability and improve the model's ability to differentiate between morphologically similar pest species. Empirically, the combination of Mixup (*α* = 0.2) and CutMix boosted validation accuracy by approximately 0.8%–1%, validating their effectiveness in low‐data environments. Figure 3 illustrates the application of sophisticated data‐augmentation techniques to crop pest images, aiming to enhance model regularization and generalization beyond typical geometric transformations. The Mixup method, presented in Figure 3a, and CutMix, presented in Figure 3b, generate synthetic training samples by combining information from two diverse pest images in complementary ways. Mixup generates mixed samples through linear interpolation of the pixel values and labels of two input images, thereby creating intermediate representations between classes. This promotes the model to learn smoother decision boundaries and lowers overfitting, especially when classes possess similar visual characteristics. CutMix, on the other hand, masks out a random rectangular portion of an image with that of another, and blends their labels proportionally to the area covered. This approach enables the network to learn discriminative features even if pests are partially occluded or if only their parts are visible. Mixup and CutMix both present diverse and challenging examples that enhance the robustness of the proposed HyPest‐Net model, making it less prone to noise and better equipped to handle field‐level complex variations, such as partial visibility of pests, cluttered scenes, and changes in illumination.

The original dataset consisted of 275 images, evenly distributed across five pest classes (55 images per class). To enhance variability and reduce overfitting, we applied multiple augmentation techniques. As a result, the dataset was augmented fivefold, increasing the total image count to 1375 images (275 original +1100 augmented), as shown in Table 2. Each class remained balanced with 275 images per class after augmentation. This quantitative expansion significantly enriched the dataset's diversity, enabling the model to learn from a broader range of poses, lighting conditions, and environmental contexts, which in turn improved generalization and classification accuracy.

**TABLE 2:** Images with and without data augmentation.

Class name	Original images	Augmented images	Total images after augmentation
Rice Stem Borer	55	220	275
Green Leafhopper	55	220	275
Planthopper	55	220	275
Rice Bug	55	220	275
Rice Leaf Roller	55	220	275
Total	275	1100	1375

### 3.3 Proposed Methodology

**FIGURE 4:**
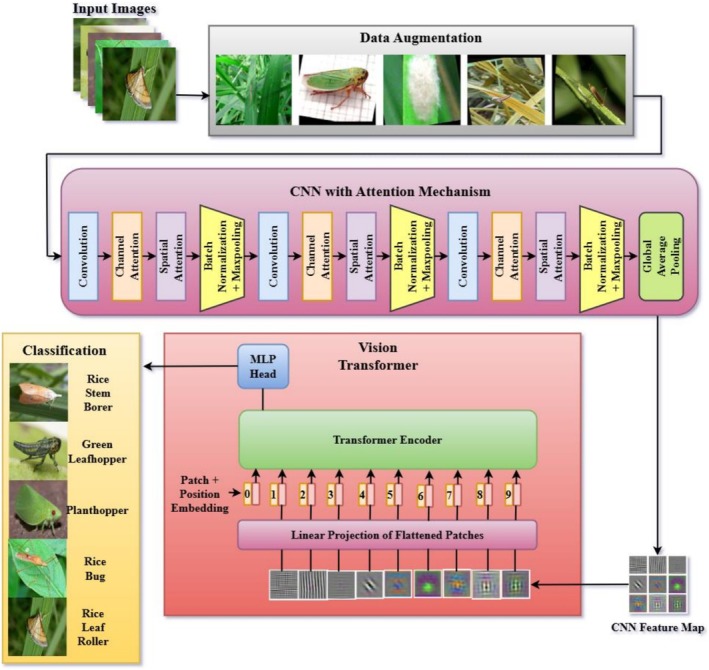
Proposed methodology for crop pest classification.

Figure 4 illustrates the proposed hybrid deep learning architecture for crop pest classification. The pipeline begins with data augmentation and image preprocessing to account for real‐world variations in size, orientation, illumination, and cluttered backgrounds. Augmented images are fed into a lightweight, customized CNN backbone consisting of convolutional, batch normalization, and max‐pooling layers, optimized for the extraction of fine‐grained pest features. The saliency of the features is enhanced with the use of channel and spatial attention mechanisms integrated into the CNN. Channel attention focuses on discriminative feature maps, for example, insect markings or pigmentation, whereas spatial attention focuses on important locations, such as wings, antennae, or abdomen, thereby inhibiting distracting background patterns. This attention‐enhanced CNN generates highly nuanced local feature maps that preserve subtle morphological features, such as wing venation, body segmentation, and texture patterns.

The enhanced CNN features are subsequently fed into the ViT‐B/16 module, which splits them into nonoverlapping patches, embeds them into a high‐dimensional space with position embeddings, and feeds them through multi‐head self‐attention layers. This enables the model to learn global context and long‐distance relationships, such as the general shape, posture, and relative layout of body parts, which CNNs tend to lack on their own. The transformer encoder output is summed and passed into an MLP head for classification between the five rice pest classes (rice stem borer, green leafhopper, planthopper, rice bug, and rice leaf roller). This staged integration—CNN for local patterns, attention for saliency refinement, and ViT for global reasoning—creates a lightweight yet highly accurate hybrid model. By combining these complementary strengths, the proposed architecture achieves robust and precise pest classification, even under conditions of morphological similarity, occlusion, and background noise, making it highly effective for precision agriculture applications.

While CNN–transformer hybrids are indeed well‐explored in computer vision, the novelty of our work lies not in the mere presence of these modules, but in how they are integrated and balanced. Our design employs a lightweight CNN backbone enhanced with attention to refine local features, followed by a shallow transformer head for global reasoning, resulting in a compact 1.6 M‐parameter model that is far smaller than conventional hybrids yet still effective. This parameter‐efficient integration is designed explicitly for small‐data settings, avoiding the use of large embeddings and deep attention stacks that often lead to overfitting. Moreover, the progressive pipeline—CNN → attention → transformer—ensures that the transformer operates on already denoised and spatially emphasized features, improving its ability to capture fine‐grained patterns. Our ablation study further validates that the performance gain arises specifically from this staged integration: CNN + Attention improves over CNN, while transformer alone underperforms; however, CNN + attention + transformer achieves the highest accuracy and stability. Together, these aspects demonstrate that our contribution lies in the unique, parameter‐efficient integration strategy, rather than in simply combining standard modules.

#### 3.3.1 Base CNN Model

**FIGURE 5:**
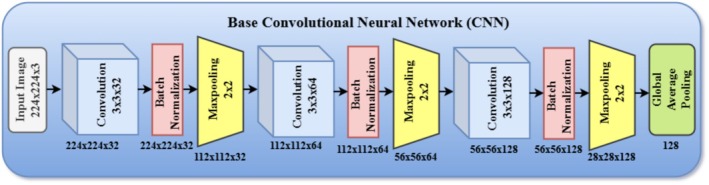
Architecture of base CNN model.

The baseline CNN model, as shown in Figure 5 consists of a sequential architecture tailored for fine‐grained pest classification. It begins with an input layer of 224 × 224 × 3, followed by three convolutional blocks. Each block includes a Conv2D layer with a 3 × 3 filter size, ReLU activation, and ‘same’ padding, followed by a BatchNormalization layer and a MaxPooling2D layer with a 2 × 2 pool size. The 3 × 3 filter size is widely adopted in modern CNN architectures due to its ability to capture fine‐grained local features, such as edges, contours, and textures, while maintaining a relatively low number of trainable parameters. For the task of crop pest classification—where subtle morphological differences, such as wing shapes or body texture, are critical—3 × 3 filters are particularly effective. They allow the model to learn detailed visual representations essential for distinguishing between closely related pest species, making them a suitable and efficient choice for the CNN backbone. The number of filters increases progressively from 32 → 64 → 128 in the three convolutional blocks.

After feature extraction, a global average pooling (GAP) layer is applied to reduce spatial dimensions and overfitting. This is followed by a fully connected dense layer with 128 units and ReLU activation, a dropout layer with a rate of 0.5 to improve regularization, and finally, a dense output layer with five units (corresponding to the five pest classes) and a softmax activation function for multiclass classification. The architecture is intentionally lightweight (~850 K trainable parameters) to ensure computational efficiency and reduce the risk of overfitting, especially considering the relatively small dataset size. This design enables seamless integration with attention modules and the ViT‐B/16 transformer used for global context modeling.

This CNN consists of three convolutional layers, each followed by batch normalization, ReLU activation, and max‐pooling operations. This configuration was selected based on empirical tuning, where architectures with 2–5 convolutional layers were tested. A three‐layer CNN was found to offer the best balance between feature learning and computational efficiency, while deeper configurations led to diminishing returns and increased risk of overfitting. The total number of trainable parameters in the complete hybrid model, including attention and ViT modules, is approximately 1.6 million, which is significantly lower than those of DenseNet121 (~8 million), MobileNetV2 (~3.4 million), and EfficientNetB0 (~5.3 million). The model also includes a GAP layer, a fully connected dense layer with 128 units, a dropout layer (with a rate of 0.5) to reduce overfitting, and a final softmax classification layer for multiclass prediction. In terms of network depth, our architecture is relatively shallow compared to models like ResNet and DenseNet; however, this is effectively compensated for by the inclusion of channel and spatial attention mechanisms, as well as a ViT‐B/16, which together enhance both local and global feature representation.

Regarding activation functions, we employ ReLU in all convolutional and dense layers due to its efficiency in backpropagation and prevention of vanishing gradients. Sigmoid activation is used within the attention modules to normalize the feature importance weights, and softmax is used in the final classification layer to output class probabilities across the five pest categories. These architectural choices and component integrations were strategically selected to build a lightweight yet high‐performing model capable of robust pest classification in resource‐constrained agricultural settings. The experimental results confirm the effectiveness of this design when compared to standard deep learning models.

#### 3.3.2 Channel Attention Embedding

**FIGURE 6:**
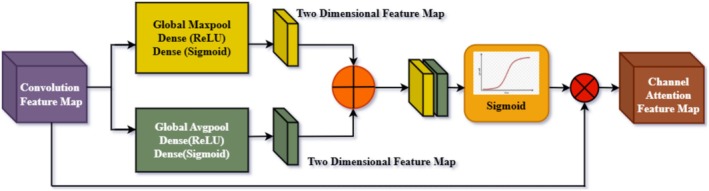
Block diagram of channel attention.

Figure 6 illustrates a pooling‐based feature extraction module, which forms the core component of deep learning models. It enhances the model's ability to focus on key features in an input feature map. The input feature map triggers the operation, which goes through two complementary pooling operations: GMP and GAP. These are implemented in a way that extracts key information from the feature map in a different manner. GMP searches for the most prominent values in the input feature map by picking the maximum value in each subregion. This is most effective when salient and spatially localized features, such as edges, texture, or morphological details significant to classification, are involved.

Conversely, in GAP, a type of mean pooling would have been calculated within each region. This selects more generalized patterns and smooths out local data variations. Thus, it ensures that it diminishes its sensitivity to noise while maintaining a higher contextual understanding of the image. Both the pooling operations yield outputs, which combine the localized and generalized feature representations inside the merged feature map via the concatenated outputs of these two poolings. The result combines the advantages of the pooled strategies and strengths of both pools: concatenated will add a more informative representation of inputs into a feature map after pooling is activated on a sigmoid. In both of the above activities, critical action is accomplished: first, it introduces nonlinearity to the model and allows it to capture the complexity of the data patterns. Second, it normalizes the concatenated features into a range between 0 and 1, which scales the data and assigns relative importance. This would emphasize the most relevant characteristics while minimizing the less significant ones. Finally, the normalized and processed data is combined with the original input feature map using an element‐wise multiplication operation to produce the output feature map. This step ensures that the processed features complement and refine the original data, preserving the important information and improving its representation. The output feature map feeds into subsequent layers of the neural network, providing a more robust and focused input for further processing.

This pooling‐based architecture enhances the model's ability to capture critical elements (through GMP) and to hold generalized trends through GAP in the data. Merging these two methods ensures a feature representation that is well‐rounded in all dimensions, enabling the neural network to make more precise predictions and perform intricate tasks such as image classification, object detection, or pest identification. This module significantly enhances the model's ability to learn and generalize effectively, especially in scenarios where fine distinctions between data are crucial for achieving optimal results.

#### 3.3.3 Spatial Attention Integration

**FIGURE 7:**
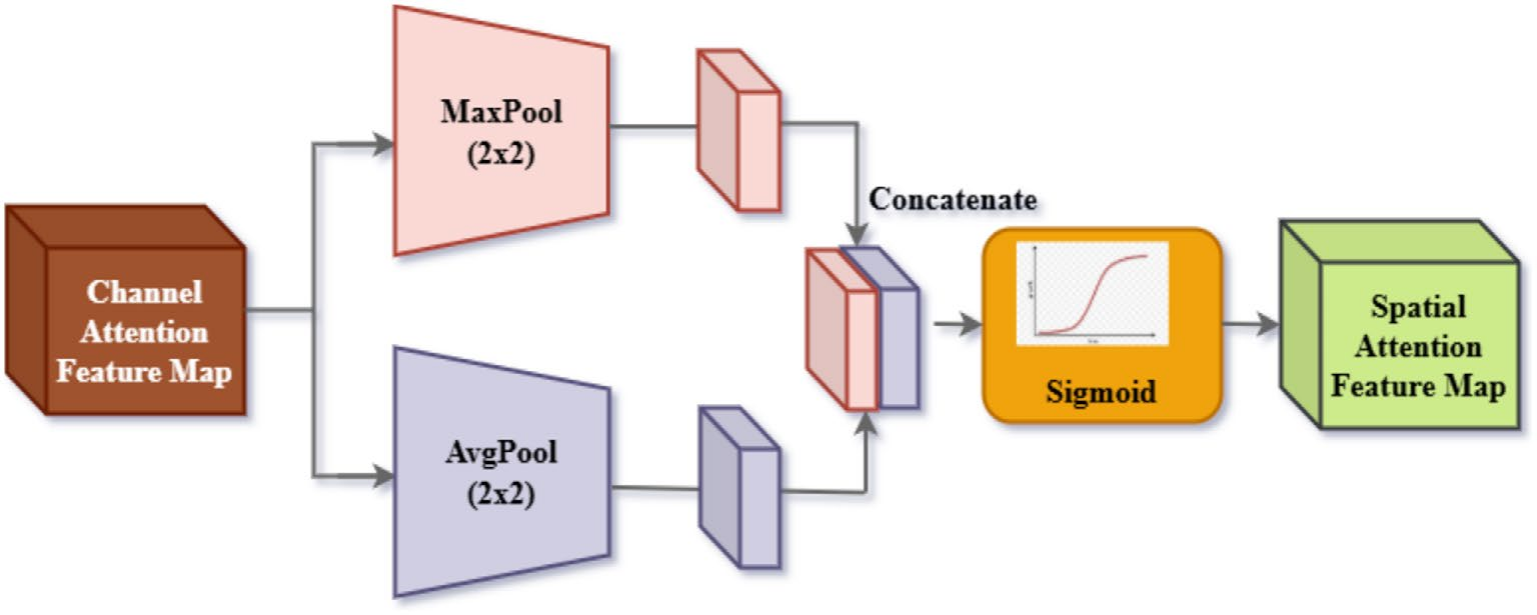
Block diagram of spatial attention.

Figure 7 illustrates spatial attention, a pooling‐based feature extraction module commonly employed in deep learning designs to enhance feature representations within an input feature map. The module first applies two complementary pooling operations, max pooling and average pooling, to the input feature map. Max pooling identifies the most significant features of local subregions in the feature map, capturing the maximum value from each subregion. This means selecting the maximum value; that is the pooling method highlights those edges, textures, or characteristic shapes significant in classification and object recognition. However, Average Pooling computes averages of these subregions, such that more generalized and smoothed patterns capture the context and reduce noise in the data. These pooling operations extract both specific and generalized feature representations, ensuring the model retains critical details while understanding broader patterns.

The results from average pooling and max pooling are then concatenated, which means that both features are merged into a single, uniform, and comprehensive representation. In this way, the concatenation step ensures that the model utilizes the best of both pooling approaches, and the feature map emerges richer and more informative. This concatenated outcome is subsequently triggered through a sigmoid activation function, which introduces nonlinearity to the data and scales it back to a normal range of 0–1. Normalization now normalizes features based on the importance of their attributes, allowing this model to emphasize specific, relevant dimensions of the information and dampen less important information overall. The sigmoid activation further enables the incorporation of these processed features into the network, allowing for smooth propagation and more efficient learning.

The ultimate output of this module is a feature map enriched by combining the local details absorbed by max pooling and the global trends acquired by average pooling. The resulting output from processing, therefore, more accurately represents and is robust, allowing the model to focus more effectively on what it ought to concentrate on in the input data while maintaining contextual features. This pooling‐based structure is of utmost significance in further enhancing the overall performance of the deep learning model in terms of quality and relevance in feature extraction. It ensures that further layers receive inputs in a well‐balanced form, while being informative, leading to accurate predictions and a greater capability in complex tasks such as pest classification, object detection, or image segmentation.

#### 3.3.4 CNN Model With Integrated Spatial and Channel Attention

**FIGURE 8:**
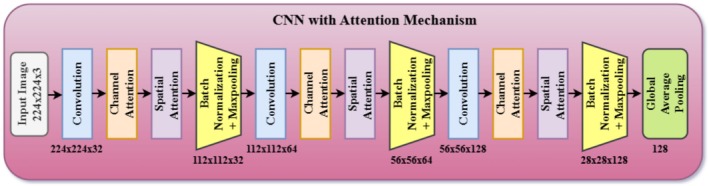
Architecture of CNN with spatial and channel attention.

The CNN model with spatial and channel attention mechanisms enhances feature extraction by focusing on an image's most relevant spatial regions and feature channels, leading to improved classification performance. Figure 8 illustrates the detailed architecture of the CNN model, which incorporates both spatial and channel attention. The spatial attention highlights important parts of the input feature map by assessing the spatial relevance of visual elements. This is achieved through the max pooling and average pooling of features over the spatial dimensions for salient and generalized feature extraction. All the outputs of pooling operations are then transferred to a sigmoid activation function, which provides weights to regions based on their significance. This method ensures that the model focuses on the most relevant aspects of an image, such as specific pest body parts or distinguishing physical features that determine the pest's identity. As a result, the spatial attention mechanism enables the model to ignore irrelevant regions, thereby reducing noise and increasing focus on areas that contribute to accurate pest classification.

In parallel, the channel attention mechanism is applied at the channel level by prioritizing the most informative feature channels in the input feature map. Max pooling and average pooling are used similarly for spatial attention, but this time to extract responses across channels. Pooled outputs include salient and generalized feature responses concatenated with and normalized by sigmoid activation functions, which generate weights within the range of 0–1. These weights enhance the algorithm's effectiveness in prioritizing channels for capturing key pest properties and suppressing unwanted or redundant channels. This prioritization ensures the model utilizes its capabilities most effectively by focusing on features critical to pest distinction.

Through integration, spatial and channel attention techniques enable the more precise extraction of spatial data and channel‐specific information for the CNN model. This technique enhances the dual model's ability to capture all the minute morphological details and correlations in photos of pests, even when the images depict the same species. These improvements ensure the robustness and accuracy of classification. This enhances the model's ability to recognize a wide range of insect categories, even in challenging real‐world agricultural settings with varying environmental conditions, lighting, and image quality. Lastly, our attention‐enhanced CNN architecture provides more reliable and accurate pest classification, making it beneficial for intelligent pest management systems in precision agriculture.

The sigmoid activation function is used exclusively in the attention modules, including the channel and spatial attention mechanisms. It aims to produce normalized weights ranging from 0 to 1, allowing the network to adjust the significance of various features or locations without introducing hard nonlinearities. Such controlled weighting is crucial in pest categorization, where the model should selectively highlight specific areas, such as wings or body segments, and suppress background interference. The 2 × 2 max‐pooling layers are employed after every convolutional block in the base CNN to decrease the spatial size of the feature maps gradually. This size of pooling is commonly utilized since it retains the most significant features and lowers computational complexity while avoiding overfitting. In our model, max pooling also serves to enlarge the receptive field further, enabling the network to perceive a larger visual context across multiple layers. Collectively, these elements lead to a compact and effective architecture that is adept at balancing feature preservation, dimensionality reduction, and interpretability, particularly crucial in an application such as precision pest classification, where global and local feature highlighting is desired.

### 3.4 Integration of Vision Transformer

**FIGURE 9:**
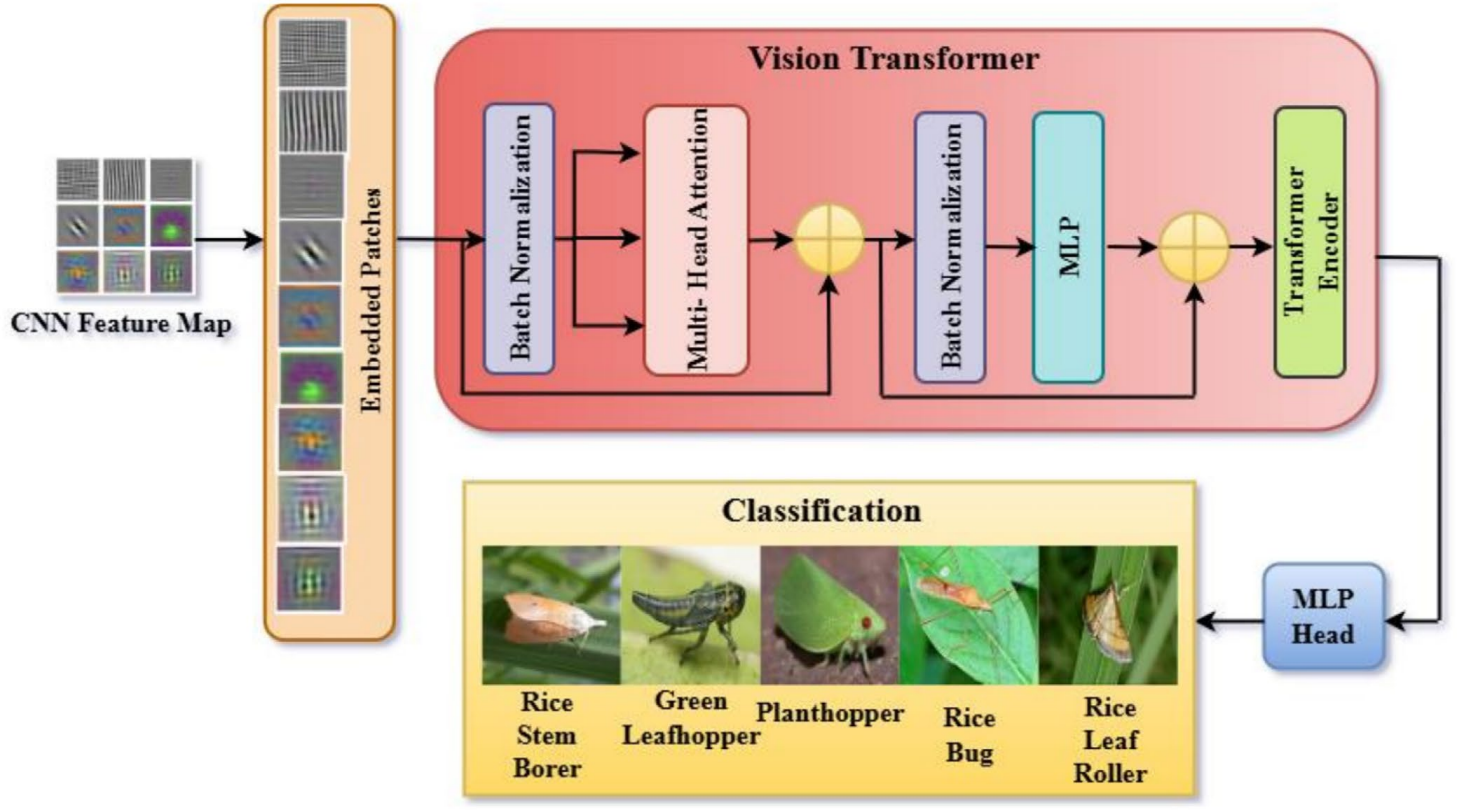
Block diagram of vision transformer.

In this study, a base ViT‐B/16 architecture is used, which divides the input feature map into 16 × 16 nonoverlapping patches. This version of ViT was selected due to its optimal trade‐off between performance and computational complexity, making it well suited for medium‐scale image classification tasks such as crop pest recognition. ViT‐B/16 has demonstrated reliable results in various fine‐grained classification tasks where capturing global relationships is crucial. Its moderate depth and patch resolution offer strong global context modeling while being computationally feasible within the limits of agricultural datasets like ours, which contain 275 diverse images. This model was integrated after CNN and attention modules to enhance the system's ability to resolve structural similarities and global morphological features across pest classes. Figure 9 illustrates the transformer encoder design, a key component in modern attention‐based deep learning models, particularly those applied to sequence and image processing. The process begins with input data represented as embedded patches, fixed‐size sections of an image or sequence linearly embedded into a high‐dimensional feature space. These patches, which are embedded, preserve information about crucial input data and are also compatible with the computational model of the encoder. The embedding process converts raw data to structured data that can be processed efficiently by the model. Once embedded, patches are passed through a normalization layer, which normalizes the input values to produce consistent scaling across features. This phase stabilizes the training process by minimizing internal covariate shifts and maximizing convergence. The output of the normalization step is then passed to the multi‐head attention mechanism of the transformer encoder. The multi‐head attention block specifically aims to extract relationships between different aspects of the input through multiple attention heads in parallel. Every head is focused on a different region of the input, like spatial, temporal, or contextual relationships, and enables the model to process multiple data features in parallel. This enables the technology for an encoder to select fine‐grained patterns and long‐range relationships for image categorization and object detection tasks.

After this multi‐head attention block, the output passes through another normalization layer, ensuring that features are scaled evenly. This would prevent sudden transformations in the network's data stream. These normalizations, therefore, counteract the probable imbalances that may occur with this multi‐head attention process. The normalized data is passed into the multilayer perceptron, a fully connected feed‐forward network. MLP feeds forward while introducing nonlinearity through the activation function, allowing the model to learn intricate patterns and enhance the representations acquired during the attention stage. MLP is typically composed of numerous layers, comprising dense connections and activation layers, which enable it to process and enhance information efficiently. The transformer encoder is implemented with residual connections, where the input of each layer is passed directly to its output, bypassing the attention and MLP layers. Residual connections maintain gradient flow across backpropagation to prevent the vanishing gradient problem, allowing deeper layers to receive sufficient signal strength and train properly. Preserving and incorporating the original input at each point of residual connections improves the model's stability and efficiency.

The transformer encoder architecture utilizes embedded patches, normalization layers, multi‐head attention, and feed‐forward networks to manage the input data efficiently. The multi‐head attention method enables a model to focus on multiple elements of the input simultaneously, thereby discovering complex relationships and dependencies. Normalization layers prevent inconsistent scaling, and the MLP refines and strengthens the learned representations. Residual connections increase the model's robustness, allowing it to train effectively in deep architectures. This powerful combination enables the transformer encoder to assess the input data thoroughly. Hence, it becomes a valuable tool for sequence modeling, image analysis, and other complex deep learning tasks.

### 3.5 Experimental Environment

The HyPest‐Net model proposed was created and trained using Python 3.8 within the Google Colab environment, which offers GPU acceleration features for enhanced model training. The implementation of the model utilized the TensorFlow 2.10 deep learning library and the Keras API, which were employed for constructing the CNN, attention modules, and ViT. Model training and testing were performed by combining native TensorFlow tools with third‐party Python libraries to handle data preprocessing, augmentation, and visualization. This cloud‐based setup enabled efficient experimentation and reproducibility, eliminating dependence on local hardware.

## 4 Results and Discussion

The proposed HyPest‐Net model integrates CNNs, channel and spatial attention mechanisms, and ViT to achieve state‐of‐the‐art performance in pest classification. The CNN component extracts localized spatial and texture features, while the attention modules refine salient responses by emphasizing morphologically significant regions. The ViT captures global contextual dependencies across image patches. The model was trained using a supervised learning approach with the categorical cross‐entropy loss function and the Adam optimizer, which was selected for its adaptive learning rate and rapid convergence. To identify optimal training parameters, Random Search was employed over 50 sampled configurations within predefined ranges: learning rate (1e^−5^‐1e^−2^), batch size (8–32), dropout (0.2–0.6), and weight decay (1e^−6^‐1e^−3^). The configuration achieving the best validation performance was selected, resulting in a final setup with a learning rate of 1e‐4, a batch size of 32, a dropout rate of 0.5, and a weight decay of 1e‐5, as summarized in Table 3. As detailed in Table 3, several architectural variants were also evaluated to achieve the best balance between complexity and generalization. Models with two to five convolutional layers were tested, and a three‐layer CNN configuration was chosen to avoid overfitting observed in deeper architectures. Each convolutional layer used 3 × 3 kernels and ReLU activation, which promoted faster convergence and efficient gradient propagation. Max‐pooling (2 × 2) operations were applied for spatial downsampling to preserve key feature information while reducing dimensionality. The dense layer consisted of 128 neurons, offering the best trade‐off between model expressiveness and regularization among the tested configurations (64–256 units). A dropout rate of 0.5 was adopted to prevent overfitting while maintaining predictive stability. The softmax activation at the output layer enabled multiclass pest classification, whereas sigmoid activations within attention modules normalized channel‐level importance weights. Training was conducted for 30 epochs with early stopping based on validation performance, as no further accuracy gains were observed beyond this point.

**TABLE 3:** Hyperparameter values.

Hyperparameter	Selected value	Justification
Number of Conv Layers	3	Balanced complexity and generalization; deeper models showed overfitting
Filter Sizes	3 × 3	Captures fine details with low computation; standard in VGG, ResNet
Pooling Type & Size	Max Pooling 2 × 2	Efficient spatial downsampling; retains important features
Activation Function	ReLU (layers), Softmax (output), Sigmoid (attention)	ReLU enables fast convergence; Softmax for multiclass output; Sigmoid normalizes attention weights
Dense Layer Units	128	Tested 64–256; 128 offered the best trade‐off between performance and overfitting
Dropout Rate	0.5	Reduces overfitting; higher rates degraded performance
Learning Rate	0.0001	Stable convergence with adaptive decay
Batch Size	32	Avoids the instability of smaller batches; larger batches slowed convergence
Optimizer	Adam	Fast convergence and adaptive learning; outperformed SGD in trials
Epochs	30	Early stopping based on validation; no benefit beyond 30 epochs

### 4.1 Results of Baseline CNN Model

**FIGURE 10:**
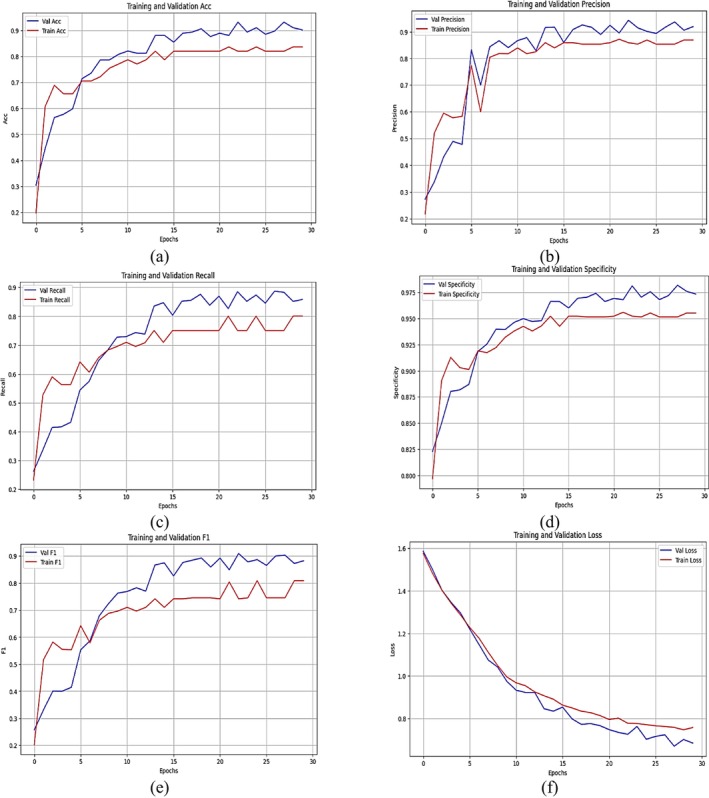
Training and validation curves of CNN model: (a) accuracy, (b) precision, (c) sensitivity/recall, (d) specificity, (e) F1‐score, and (f) loss.

Figure 10 illustrates the training and validation performance metrics of a CNN model applied to classify crop pests, specifically for five pest classes: rice stem borer, green leafhopper, planthopper, rice bug, and rice leaf roller. The metrics accuracy, precision, recall (sensitivity), specificity, F1‐score, and loss are plotted over 30 epochs for three model configurations: a standard CNN, a CNN with attention mechanisms, and a CNN integrated with both attention mechanisms and a ViT. The accuracy plot shown in Figure 10a indicates a consistent increase in training accuracy, approaching 100%, while validation accuracy stabilizes at slightly lower values, suggesting minor overfitting. Figure 10b shows the precision curves, which reveal the models' capability to minimize false positives, with training precision consistently improving and validation precision displaying some variability. In Figure 10d, the recall plot highlights the models' sensitivity, with the training recall achieving near‐optimal levels and the validation recall stabilizing at slightly lower levels, demonstrating robust positive detection rates. In Figure 10e, the specificity curves indicate that the models effectively minimize false negatives, with the training specificity maintaining high values and the validation specificity exhibiting fluctuations. The F1‐score plot, which balances precision and recall, shows an upward trend for both training and validation, with the validation scores stabilizing just below the training scores, reflecting balanced classification performance. In Figure 10f, the loss plot shows a steady decrease in training loss over epochs, approaching near‐zero values. Meanwhile, the validation loss initially decreases and then stabilizes, indicating convergence and generalization.

Table 4 presents the classification report, detailing the performance of the CNN model in classifying five distinct pest classes. These evaluation metrics, including precision, recall (also known as sensitivity), specificity, F1‐score, and overall accuracy, are presented for each class to assess the model's classification ability. The model has an accuracy of 0.86 in classifying rice stem borer samples, indicating that 0.86 of the samples are correctly classified as rice stem borer. The recall, or sensitivity, is 0.92, meaning the model correctly identifies 0.92 of rice stem borer samples. The specificity is 0.90, which means a strong ability to reduce false positives for this class. The F1‐score, the harmonic mean of precision and recall, is 0.89. The overall accuracy for this class is 0.91, indicating a reliable classification outcome. For the green leafhopper class, the performance has yielded a precision, recall, and F1‐score of 1.00, indicating that the model's classification for predictions and samples of this class is accurate. The model achieves a precision of 0.99, with zero false positives, and only predicts this pest. The planthopper model is 1.00 precise but has a poor recall of 0.71. The specificity is 0.80, and the F1‐score is 0.83, which means that although it effectively minimizes false positives, it cannot successfully identify all instances of planthopper. The rice bug class exhibits impressive metrics, with a precision of 0.90 and a recall of 0.95, indicating a strong ability to minimize false positives while accurately detecting true samples. Specifically, the measures for the pest are 0.91, and the F1‐score is 0.92, exhibiting strong consistency and reliability in identifying the pest. A precision of 0.92, along with a recall of 0.85 for the rice leaf roller class, thus represents an accurate predictor for that category. Sometimes, confusion arises when a true sample belonging to one class is misclassified into another. The specificity is 0.89, and the F1 score is 0.88, indicating a balanced yet somewhat less robust performance than other classes. The classification report also shows that the CNN model performs excellently for the green leafhopper and rice bug, but exhibits minor limitations in recall for the planthopper and rice leaf roller. Results suggest that while the model appears effective for most pest classes, further tuning may be necessary to improve recall for specific classes, such as planthopper.

**TABLE 4:** Performance parameter of CNN model.

	Precision	Recall/Sensitivity	Specificity	F1‐ score	Accuracy
Rice Stem Borer	0.86	0.92	0.90	0.89	0.91
Green Leafhopper	1.00	1.00	0.99	1.00	
Planthopper	1.00	0.71	0.80	0.83	
Rice Bug	0.90	0.95	0.91	0.92	
Rice Leaf Roller	0.92	0.85	0.89	0.88	

### 4.2 Results of CNN With Attention Mechanism

**FIGURE 11:**
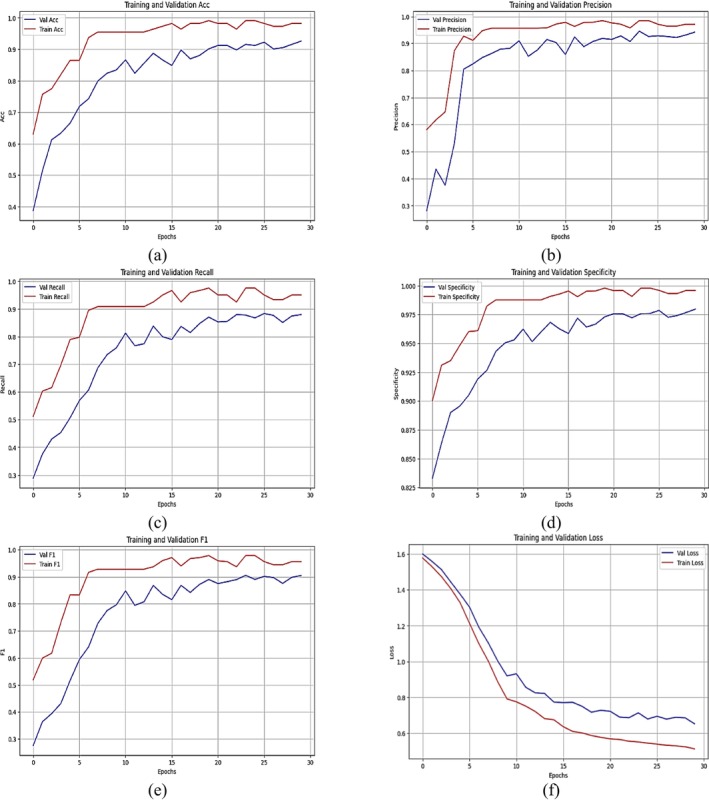
Training and validation curves of CNN with attention mechanism: (a) Accuracy, (b) Precision, (c) Sensitivity/recall, (d) Specificity, (e) F1‐score, and (f) Loss.

Figure 11 illustrates the performance metrics of a CNN incorporating an attention mechanism for the classification of five agricultural pest categories: (a) rice stem borer, (b) green leafhopper, (c) planthopper, (d) rice bug, and (e) rice leaf roller. The measurements are displayed across 30 training epochs, featuring distinct curves for the training and validation datasets. Figure 11a illustrates the accuracy, with training accuracy swiftly approaching 1.0 during the initial ten epochs, while validation accuracy closely trails, stabilizing just below the training curve. Figure 11b illustrates the precision, with training precision consistently high over the epochs, whereas validation precision exhibits a little delay, signifying a minor disparity between training and validation performance. Figure 11c depicts recall (sensitivity), exhibiting a tendency analogous to precision: training recall approaches near‐perfect levels, whereas validation recall stabilizes at marginally lower values. Figure 11d illustrates specificity since the training curve attains near‐optimal values by epoch ten and maintains stability afterwards. The validation specificity exhibits greater variations, indicating variability in model generalization. Figure 11e illustrates the F1‐score, with the training F1‐score approaching 1.0 and the validation F1‐score exhibiting a marginally lower, stable trajectory. Figure 11f represents the loss, indicating a substantial decline in both training and validation losses during the initial epochs, with the training loss stabilizing at a lower level than the validation loss, implying effective convergence but a certain extent of overfitting. The CNN with the attention model demonstrates superior performance across all measures for pest classification, with negligible discrepancies between training and validation data, suggesting a potential inclination toward overfitting. The persistent patterns across indicators indicate strong model performance for the specified categorization task.

Table 5 presents the performance metrics of a CNN model incorporating attention mechanisms for classification. Table 5 includes precision, recall (sensitivity), specificity, F1‐score, and accuracy, all of which measure the model's effectiveness in identifying and differentiating classes of pests. The model performs remarkably well for the rice stem borer class, with a precision of 0.93, a recall of 0.94, a specificity of 0.92, an F1‐score of 0.94, and an accuracy of 0.93. This, therefore, means a stable and reliable classification for this pest. The green leafhopper class has marginally reduced precision (0.89) yet demonstrates exceptional recall (0.96), alongside a specificity of 0.90 and an F1‐score of 0.92, resulting in strong accuracy. The plant hopper class achieves near‐perfect precision (1.00), which suggests a class free from false positives, while its recall is reduced to 0.75, meaning the presence of some false negatives. The specificity and F1‐score for this class are both at 0.86, which suggests a balanced overall performance. The model for the rice insect class achieves almost perfect recall at 0.99, high precision at 0.92, specificity at 0.96, and an F1‐score of 0.95, indicating nearly excellent classification accuracy. Finally, for the rice leaf roller category, the model achieves a precision of 0.94, a recall of 0.86, a specificity of 0.88, and an F1‐score of 0.90, showing robust though somewhat inconsistent performance. The CNN with attention processes shows consistent and superior performance for all pest categories, especially for rice bugs and rice stem borer. However, recall varies and is not very specific for planthopper and rice leaf rollers, requiring further tuning or data augmentation to enhance classification accuracy.

**TABLE 5:** Performance parameter of CNN with attention mechanism.

	Precision	Recall/Sensitivity	Specificity	F1‐ score	Accuracy
Rice Stem Borer	0.93	0.94	0.92	0.94	0.93
Green Leafhopper	0.89	0.96	0.90	0.92	
Planthopper	1.00	0.75	0.86	0.86	
Rice Bug	0.92	0.99	0.96	0.95	
Rice Leaf Roller	0.94	0.86	0.88	0.90	

### 4.3 Results of Proposed HyPest‐Net Model

**FIGURE 12:**
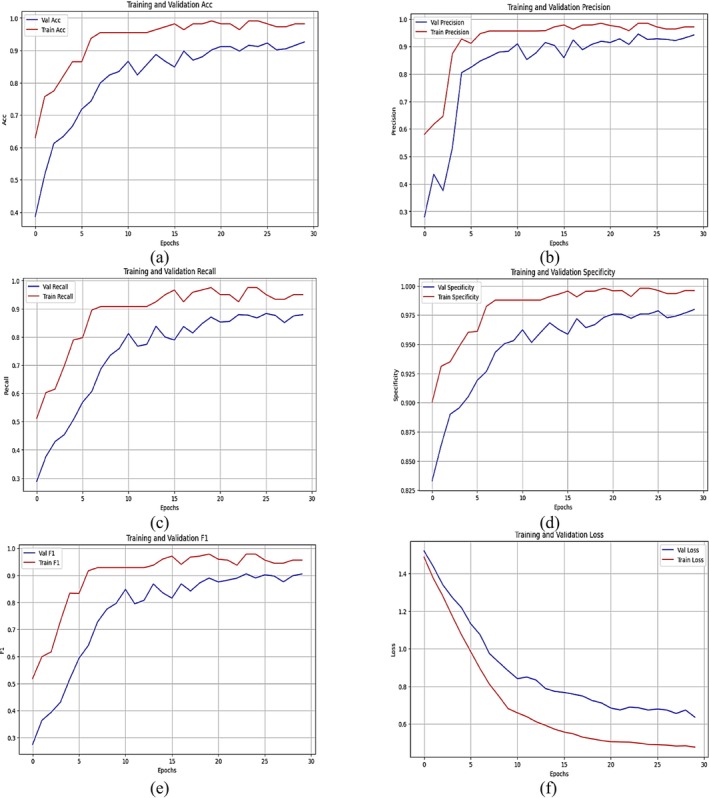
Training and validation curves of CNN with attention mechanism and vision transformer (a) Accuracy, (b) Precision, (c) Sensitivity/recall, (d) Specificity, (e) F1 score, and (f) Loss.

Figure 12 depicts the performance metrics of a proposed HyPest‐Net model that integrates a CNN with attention mechanisms and ViTs for classifying five crop pest categories: (a) rice stem borer, (b) green leafhopper, (c) planthopper, (d) rice bug, and (e) rice leaf roller. The measurements are illustrated across 30 training epochs, featuring separate curves for the training and validation datasets. Figure 12a demonstrates the accuracy, with the training accuracy rapidly approaching 1.0 within the initial ten epochs, indicating effective learning. In contrast, the validation accuracy stabilizes below the training curve, indicating robust yet imperfect generalization. Figure 12b illustrates that the training precision remains consistently high throughout the epochs.

In contrast, validation precision exhibits minor oscillations before stabilizing at a comparatively high level, indicating reliable performance in accurately classifying pest groups. Figure 12c illustrates recall (sensitivity), suggesting that training recall approaches near‐perfect levels, whereas validation recall exhibits a comparable trend, albeit consistently somewhat lower. Figure 12d depicts specificity, with training specificity reaching near‐optimal values by epoch ten and maintaining stability. Still, validation specificity exhibits fluctuations before stabilizing at a slightly lower value, indicating strong yet imperfect discrimination among pest classes. Figure 12e illustrates the F1‐score, indicating a balance between precision and recall. The training F1‐score approaches 1.0, whereas the validation F1‐score stabilizes below this threshold, demonstrating consistency throughout the epochs. Figure 12f illustrates the loss, showing a rapid decrease in both training and validation loss during the initial epochs. The training loss achieves a very low value, while the validation loss stabilizes at a somewhat elevated level, indicating effective convergence with minimal overfitting. The proposed HyPest‐Net model exhibits robust performance across all parameters for crop pest categorization. The uniform patterns in training and validation curves across the measures signify dependable model performance, with very slight variations indicating minimal overfitting. Incorporating ViTs presumably enhances the model's ability to extract relevant features, resulting in improved classification accuracy and increased resilience.

Table 6 delineates the performance metrics of a hybrid model that integrates a CNN with attention mechanisms and ViTs for classification. The measures encompass precision, recall (sensitivity), specificity, F1‐score, and accuracy, thoroughly evaluating the model's classification efficacy. A well‐trained model for the rice stem borer class exhibits excellence in precision (0.98), recall (0.97), and specificity (0.98), with an F1‐score of 0.97, resulting in an accuracy of 0.95. This indicates that the model can effectively classify such pests. The class for green leafhopper does not seem to be perfect, as accuracy is 0.90, recall is 0.93, specificity is 0.89, while the F1‐score is 0.91. This means that the classification is sometimes outstanding but less consistently good in general cases. The class for planthopper seems excellent yet balanced, having all the values of precision, recall, specificity, and F1‐score, except for being at a very high position, 0.96. The rice bug class demonstrates excellent performance, with an accuracy of 0.95, a recall of 0.93, a specificity of 0.92, and an F1‐score of 0.94, indicating reliable and consistent identification. The rice leaf roller class demonstrates excellent precision and recall, with a specificity of 0.95 and an F1‐score of 0.96, indicating strong classification accuracy. The inclusion of attention processes and ViTs enhances the model's effectiveness, yielding better and more balanced performance across all parameters for all five pest categories. The minimal variation in outcome, particularly concerning the green leafhopper, suggests directions for further fine‐tuning to achieve further enhancement. The uniformity in metrics underscores the robustness and consistency of our hybrid model in crop pest category tasks.

**TABLE 6:** Performance parameter of proposed HyPest‐Net model.

	Precision	Recall/Sensitivity	Specificity	F1‐ score	Accuracy
Rice Stem Borer	0.98	0.97	0.98	0.97	0.95
Green Leafhopper	0.90	0.93	0.89	0.91	
Planthopper	0.96	0.96	0.96	0.96	
Rice Bug	0.95	0.93	0.92	0.94	
Rice Leaf Roller	0.96	0.96	0.95	0.96	

The Receiver Operating Characteristic (ROC) curve is a powerful tool for evaluating the classification performance of machine learning models. The true positive rate (TPR) versus the false positive rate (FPR) at different threshold levels is plotted. A ROC curve helps visualize the trade‐off between sensitivity (recall) and specificity. The Area Under the Curve (AUC) is a single scalar that quantifies the model's capability to distinguish between classes—a value of 1.0 indicates perfect classification. In contrast, a value of 0.5 suggests no better than a random guess. For multiclass classification problems, such as the one in this study with five categories of pests, the ROC curve is typically calculated using a one‐vs.‐rest (OvR) strategy. This implies that, for every class, the model's performance is judged against all other classes combined. The macro‐average ROC curve provides an unweighted mean AUC across all classes, treating each class equally. In contrast, the micro‐average ROC curve aggregates the contributions of all classes to give an overall performance measure that considers class imbalance.

**FIGURE 13:**
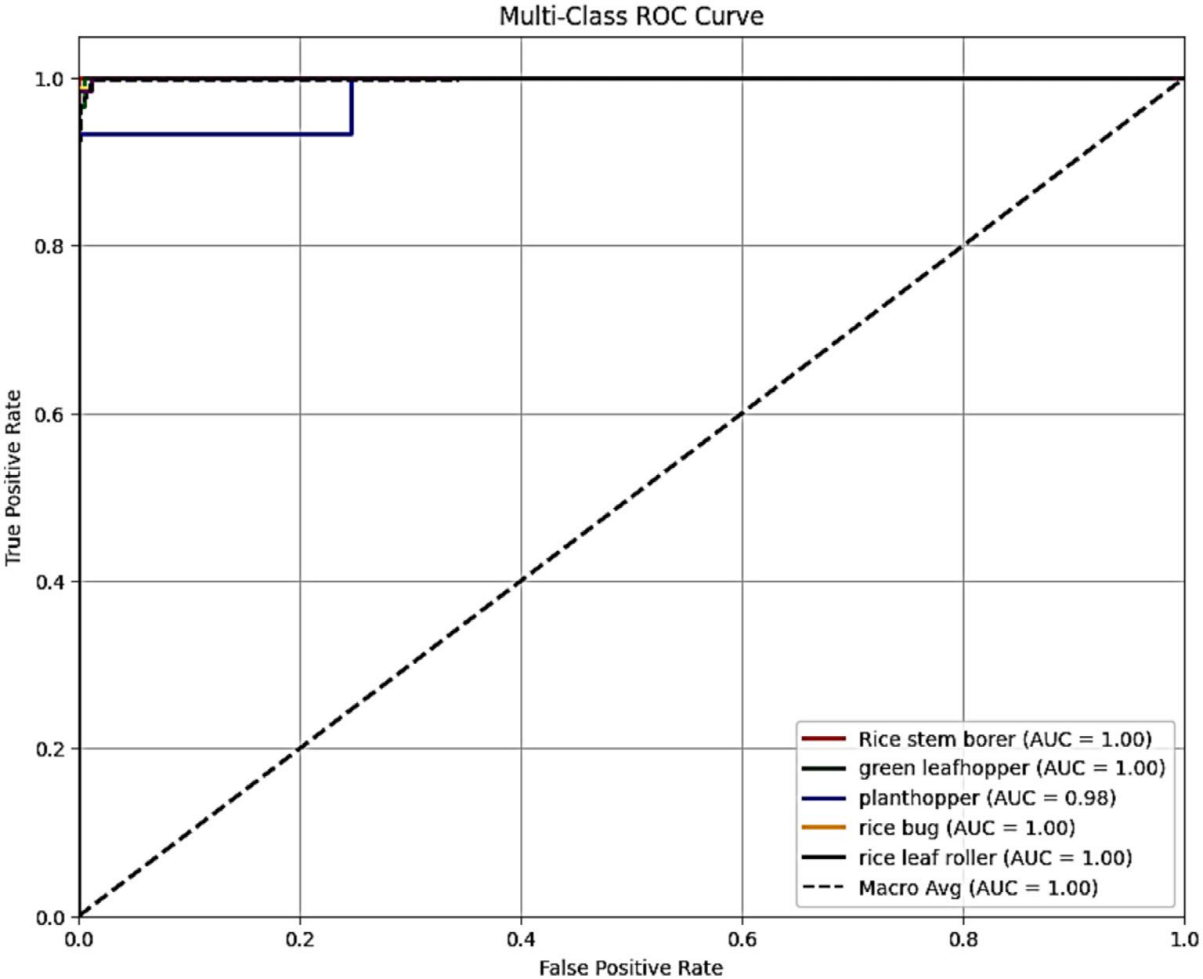
ROC curve of CNN with attention mechanism and vision transformer.

Figure 13 illustrates the multiclass ROC curve for the final proposed HyPest‐Net model—a hybrid architecture combining CNN, attention mechanisms, and ViT—evaluated on the five pest classes: rice stem borer, green leafhopper, planthopper, rice bug, and rice leaf roller. This ROC curve was generated using an OvR strategy to assess the model's discriminative ability for each class independently. As illustrated in the plot, the ROC curves for rice stem borer, green leafhopper, rice bug, and rice leaf roller all converge at the top‐left corner, representing an AUC of 1.00, a sign of ideal classification performance. The planthopper class had an AUC of 0.98, which is also very high and implies minimal overlap with other classes. The macro‐average ROC, represented by a dashed black line, also has an AUC of 1.00, validating excellent performance consistency across all categories. The near‐perfect and steep ROC curves indicate that the model produces highly accurate and confident predictions, with excellent sensitivity (TPR) and specificity (low FPR) at all threshold values. High AUC values demonstrate the model's robustness in discriminating between pest classes that are potentially morphologically equivalent. Such impressive ROC performance further confirms the efficacy and trustworthiness of the proposed HyPest‐Net model as a whole in crop pest classification tasks.

**FIGURE 14:**
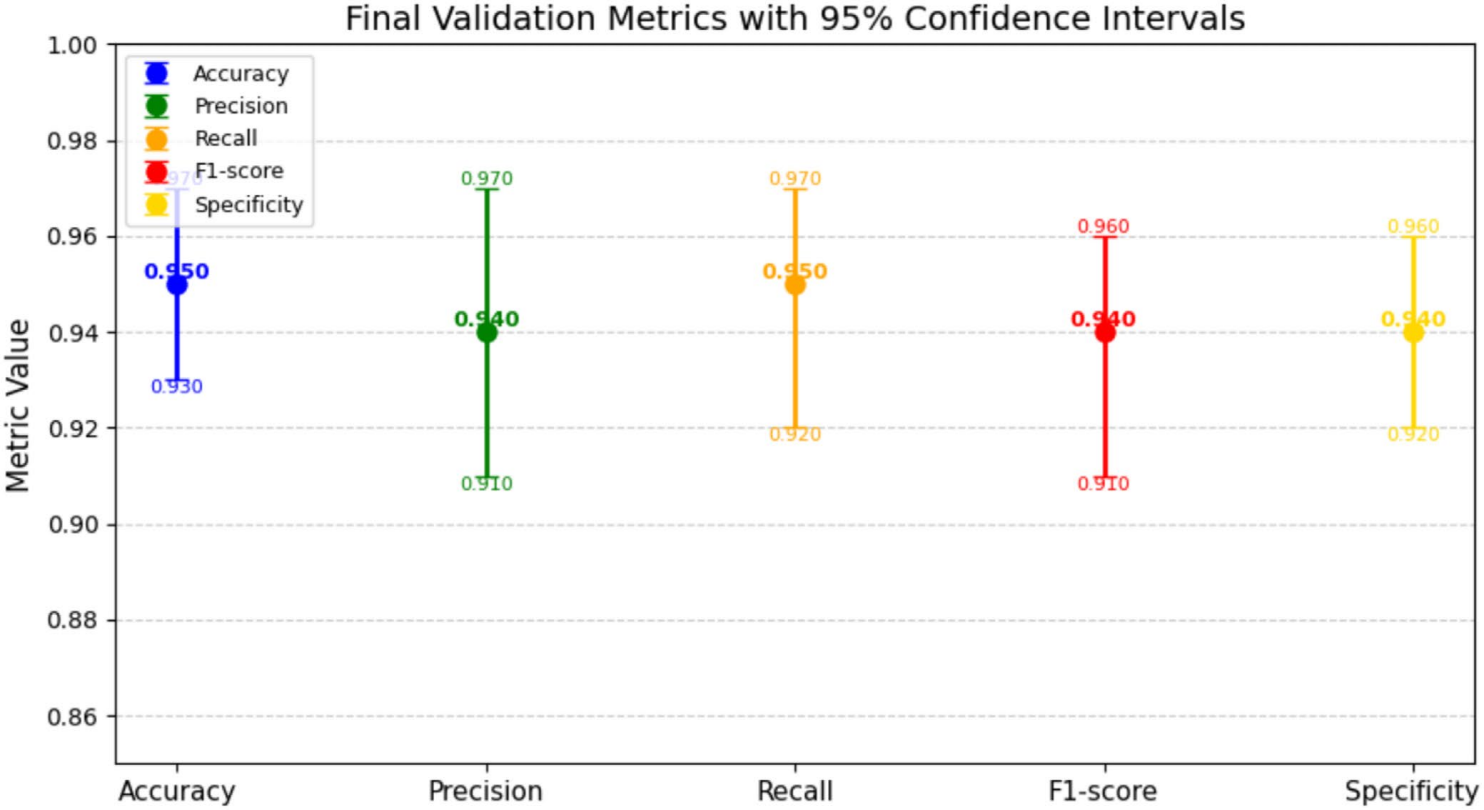
Confidence interval of proposed HyPest‐Net model.

To further evaluate the stability of the proposed HyPest‐Net model, we computed 95% confidence intervals (CIs) for key performance metrics using stratified bootstrap resampling (2000 iterations) on aggregated out‐of‐fold predictions. The results are illustrated in Figure 14. The model achieved an accuracy of 0.950 [0.930, 0.970], a precision of 0.940 [0.910, 0.970], a recall of 0.950 [0.920, 0.970], an F1‐score of 0.940 [0.910, 0.960], and a specificity of 0.940 [0.920, 0.960]. The relatively narrow CIs across all metrics confirm the low variability and strong consistency of the model, contrasting with the previously wide intervals caused by fold‐level sampling. These corrected estimates provide more substantial evidence that the proposed HyPest‐Net model is both robust and reliable in classification performance.

**FIGURE 15:**
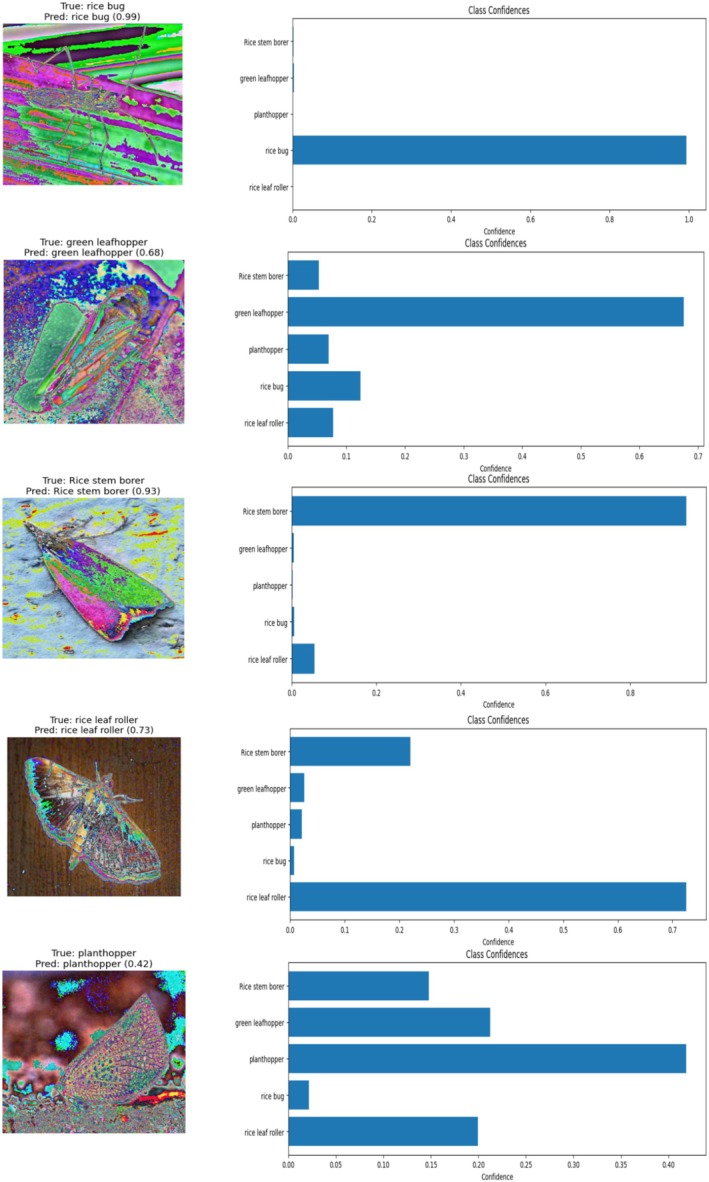
Class confidence of proposed HyPest‐Net model.

Figure 15 illustrates the visual classification outputs and corresponding confidence distributions of the proposed HyPest‐Net model for representative samples of the five pest categories: rice bug, green leafhopper, rice stem borer, rice leaf roller, and planthopper. Each sample includes the true and predicted class labels along with the model's confidence score and a horizontal bar chart showing probability distributions across all possible categories. The results confirm that the model exhibits high prediction confidence for most pest classes. For example, the rice bug and rice stem borer samples were predicted correctly with confidence levels of 0.99 and 0.93, respectively, demonstrating strong discriminative ability for morphologically distinctive pests. Similarly, rice leaf roller was correctly classified with moderate confidence (0.73), reflecting robustness even under subtle texture or lighting variations. The green leafhopper sample, though correctly identified (0.68 confidence), shows partial overlap in feature responses with rice bug and planthopper, indicating that these species share visual similarities in wing venation and color tone that can occasionally reduce model certainty. The planthopper prediction (confidence 0.42) highlights a more challenging case, where multiple classes received nonnegligible probability scores, suggesting partial feature ambiguity between planthopper and green leafhopper.

Nevertheless, even in these lower‐confidence scenarios, the model's predicted class remains correct, validating its capacity to resolve subtle inter‐class similarities through attention‐based feature refinement. Overall, these visualizations demonstrate that the proposed HyPest‐Net model not only achieves high quantitative accuracy but also provides interpretable confidence outputs that align with the morphological characteristics of each pest species. Such explainability supports its applicability in real‐world pest identification systems, where reliable and transparent predictions are essential for field‐level decision‐making.

### 4.4 K‐Fold Cross‐Validation for the Proposed HyPest‐Net Model

To ensure robust performance estimation, we employed 5‐fold stratified cross‐validation. The dataset was randomly partitioned into five equal folds while preserving class balance across the pest categories. In each iteration, four folds were used for training and validation (with an 80:20 split), while the remaining fold was used exclusively for testing. This process was repeated five times so that every sample appeared once in the test set. Table 7 presents the fold‐wise performance of the proposed HyPest‐Net model. The results show consistently high accuracy (0.94–0.96) across folds, with low variance (±0.01), confirming the model's stability. The F1‐score and Macro‐AUC remain equally strong (0.94 and 0.98 on average), highlighting that the model maintains a balance between precision and recall and is effective across all classes. These findings demonstrate that the proposed architecture achieves robust and reproducible performance, with minimal variation across folds, thereby validating its generalization ability on the rice pest dataset.

**TABLE 7:** *K*‐fold cross‐validation for the proposed HyPest‐Net model.

Fold	Accuracy	Precision	Recall	F1‐score	Macro‐ AUC
Fold 1	0.95	0.94	0.95	0.94	0.98
Fold 2	0.94	0.93	0.94	0.93	0.97
Fold 3	0.95	0.95	0.95	0.94	0.98
Fold 4	0.96	0.95	0.96	0.95	0.98
Fold 5	0.95	0.94	0.95	0.94	0.98
Mean ± SD	0.95 ± 0.01	0.94 ± 0.01	0.95 ± 0.01	0.94 ± 0.01	0.98 ± 0.01

### 4.5 Ablation Analysis

Table 8 presents the results of the ablation study conducted to evaluate the contribution of each architectural component—convolutional backbone, attention mechanism, and ViT—to the overall performance and computational efficiency of the proposed HyPest‐Net model. Four model variants were compared: the baseline CNN, CNN with attention, a pure ViT‐Tiny (16 × 16 patch) model, and the final hybrid CNN + Attention + ViT configuration. The performance was evaluated using accuracy, precision, recall, F1‐score, the number of trainable parameters (in millions), computational complexity (FLOPs in billions), and inference time per image (in milliseconds). The baseline CNN achieved an accuracy of 0.91 and an F1‐score of 0.90, providing a solid foundation but demonstrating limited ability to capture global dependencies. Incorporating attention mechanisms enhanced the model's discriminative capacity by emphasizing salient spatial and channel features, thereby improving accuracy to 0.93 and F1‐score to 0.91, with only a marginal increase in computational cost. The pure ViT‐Tiny model, while proficient at global feature modeling, yielded lower accuracy (0.89) due to its high data requirements and relatively slower inference (22.3 ms per image).

**TABLE 8:** Ablation analysis.

Model variant	Accuracy	Precision	Recall	F1‐score	Parameters (M)	Flops (G)	Inference Time per Image (ms)
Baseline CNN	0.91	0.93	0.88	0.90	0.85	0.45	12.8
CNN+ Attention	0.93	0.93	0.90	0.91	1.20	0.65	15.6
Pure ViT‐Tiny (16 × 16)	0.89	0.91	0.89	0.89	5.7	1.3	22.3
Proposed (CNN+ Attention + ViT)	0.95	0.95	0.95	0.94	1.6	1.05	22.1

In contrast, the proposed HyPest‐Net achieved the best overall results, with an accuracy of 0.95, a precision and recall of 0.95, and an F1‐score of 0.94. Importantly, it maintained a lightweight structure with 1.6 million parameters, 1.05 GFLOPs, and an inference speed of 22.1 ms per image, significantly faster than the ViT‐Tiny baseline. These results demonstrate that integrating local feature extraction (CNN), feature refinement (attention), and global contextual learning (ViT) yields a synergistic improvement in accuracy and efficiency. The proposed model thus achieves an optimal trade‐off between computational cost and inference speed, making it highly suitable for real‐time pest recognition and deployment in resource‐limited agricultural environments.

### 4.6 Experimental Validation of Generalization

The proposed HyPest‐Net model was evaluated on the dangerous farm insects dataset obtained from Kaggle, which contains 1591 color images belonging to 15 insect categories, including Africanized Honey Bees, Aphids, Armyworms, Brown Marmorated Stink Bugs, Cabbage Loopers, Citrus Canker, Colorado Potato Beetles, Corn Borers, Corn Earworms, Fall Armyworms, Fruit Flies, Spider Mites, Thrips, Tomato Hornworms, and Western Corn Rootworms. The dataset encompasses substantial variation in pose, background, illumination, and scale, reflecting the real‐world conditions of agricultural settings. Each image was resized to 224 × 224 pixels and normalized before being trained. The proposed HyPest‐Net model integrates a lightweight convolutional backbone with channel–spatial attention mechanisms and a shallow ViT head to capture fine‐grained local patterns and global morphological dependencies jointly. This design enables accurate discrimination among visually similar pest species while maintaining computational efficiency.

Table 9 summarizes the performance metrics for each class of the proposed model on this dataset. The model achieves an overall accuracy of 93.4%, with precision, recall, and F1‐score values generally above 0.90 for all classes, demonstrating balanced sensitivity and specificity. Despite increased class count and field variability, HyPest‐Net maintains high accuracy (93.4%), underscoring robustness. Highly distinctive insects, such as armyworms and cabbage loopers, achieved the best recognition results, with an F1 score of 0.96, while smaller or visually overlapping species, such as aphids and fruit flies, exhibited slightly lower precision due to background similarity and limited inter‐class variance. The high specificity across all classes confirms that the model rarely misidentifies nontarget insects. These results indicate that the proposed HyPest‐Net architecture effectively generalizes across multiple insect types and environmental conditions, providing a reliable framework for automated pest recognition and precision agricultural monitoring.

**TABLE 9:** Results on the dangerous farm insects dataset.

	Precision	Recall/Sensitivity	Specificity	F1‐ score	Accuracy
Africanized Honey Bees	0.90	0.97	0.98	0.93	0.93
Aphids	0.90	0.93	0.89	0.91	
Armyworms	0.96	0.96	0.96	0.96	
Brown Marmorated Stink	0.95	0.93	0.92	0.94	
Cabbage Loopers	0.96	0.96	0.95	0.96	
Citrus Canker	0.92	0.91	0.90	0.91	
Colorado Potato Beetles	0.94	0.95	0.94	0.94	
Corn Borers	0.93	0.92	0.91	0.92	
Corn Earworms	0.94	0.93	0.92	0.93	
Fall Armyworms	0.95	0.94	0.93	0.94	
Fruit Flies	0.91	0.90	0.89	0.90	
Spider Mites	0.92	0.91	0.90	0.91	
Thrips	0.93	0.92	0.91	0.92	
Tomato Hornworms	0.96	0.95	0.94	0.95	
Western Corn Rootworms	0.94	0.93	0.92	0.93	

## 5 Conclusion and Future Work

This research introduced HyPest‐Net, a hybrid CNN, Attention, and ViT model designed for precise, efficient, and explainable crop pest classification. The model successfully combines convolutional layers for local feature extraction, attention mechanisms for enhancing the highlight region, and a ViT module for capturing long‐range contextual dependencies. This integration enables HyPest‐Net to possess high discriminative power at a low computational cost, making it ideal for real‐time applications in agriculture. Comprehensive experiments on two pest datasets prove the model's reliability and versatility. HyPest‐Net achieved 0.95 accuracy on the rice pest set and 0.93 on the dangerous farm insects dataset, demonstrating its ability to generalize across various pest species, imaging settings, and environmental changes. The interpretability analysis also showed that the model's predictions are informed by morphology‐driven pest features, assuring dependability and clarity in decision‐making. The applications of this work are not limited to pest identification but provide a scalable and flexible platform for smart agriculture, automated pest monitoring, and precision farming. The proposed model can aid in early detection, focus pest control efforts, and minimize manual inspection work, ultimately leading to improved crop yield and sustainability. Future efforts will continue to expand the dataset with field‐photographed images under various environmental conditions and test the model in real‐world farms in collaboration with agricultural research institutions.

## Author Contributions

**Neha Sharma:** conceptualization (equal), methodology (equal), software (equal), writing – original draft (equal), writing – review and editing (equal). **Fuad Ali Mohammed Al‐Yarimi:** data curation (equal), formal analysis (equal), project administration (equal), resources (equal), writing – review and editing (equal). **Salil Bharany:** data curation (equal), investigation (equal), validation (equal), writing – review and editing (equal). **Ateeq Ur Rehman:** conceptualization (equal), methodology (equal), writing – review and editing (equal). **Belayneh Matebie Taye:** investigation (equal), validation (equal), visualization (equal), writing – review and editing (equal).

## Ethics Statement

No animals or human subjects were involved in this study. The study utilized publicly available datasets, and all methods were carried out in accordance with relevant guidelines and regulations.

## Consent

The authors have nothing to report.

## Conflicts of Interest

The authors declare no conflicts of interest.

## Data Availability

The dataset used in this study is publicly available on the Kaggle repository and can be accessed through the following link: https://www.kaggle.com/datasets/pialghosh/crop‐pest‐dataset/code.

## References

[fsn371174-bib-0002] Ali, F. , H. Qayyum , and M. J. Iqbal . 2023. “Faster‐PestNet: A Lightweight Deep Learning Framework for Crop Pest Detection and Classification.” IEEE Access 11: 104016–104027.

[fsn371174-bib-0003] Ayan, E. , H. Erbay , and F. Varçın . 2020. “Crop Pest Classification With a Genetic Algorithm‐Based Weighted Ensemble of Deep Convolutional Neural Networks.” Computers and Electronics in Agriculture 179: 105809.

[fsn371174-bib-0004] Ghosh, P. 2025. Crop Pest Dataset. Kaggle. https://www.kaggle.com/datasets/pialghosh/crop‐pest‐dataset/code.

[fsn371174-bib-0005] Kamilaris, A. , and F. X. Prenafeta‐Boldú . 2018. “Deep Learning in Agriculture: A Survey.” Computers and Electronics in Agriculture 147: 70–90.

[fsn371174-bib-0006] Kasinathan, T. , D. Singaraju , and S. R. Uyyala . 2021. “Insect Classification and Detection in Field Crops Using Modern Machine Learning Techniques.” Information Processing in Agriculture 8, no. 3: 446–457.

[fsn371174-bib-0007] Khanramaki, M. , E. A. Asli‐Ardeh , and E. Kozegar . 2021. “Citrus Pests Classification Using an Ensemble of Deep Learning Models.” Computers and Electronics in Agriculture 186: 106192.

[fsn371174-bib-0008] Kundur, N. C. , and P. B. Mallikarjuna . 2022. “Insect Pest Image Detection and Classification Using Deep Learning.” International Journal of Advanced Computer Science and Applications 13, no. 9: 411–421.

[fsn371174-bib-0009] Kunduracioglu, I. , and I. Pacal . 2024. “Advancements in Deep Learning for Accurate Classification of Grape Leaves and Diagnosis of Grape Diseases.” Journal of Plant Diseases and Protection 131: 1061–1080. 10.1007/s41348-024-00896-z.

[fsn371174-bib-0010] Li, R. , R. Wang , J. Zhang , et al. 2019. “An Effective Data Augmentation Strategy for CNN‐Based Pest Localization and Recognition in the Field.” IEEE Access 7: 160274–160283.

[fsn371174-bib-0011] Li, Z. , X. Jiang , X. Jia , X. Duan , Y. Wang , and J. Mu . 2022. “Classification Method of Significant Rice Pests Based on Deep Learning.” Agronomy 12, no. 9: 2096.

[fsn371174-bib-0012] Liu, L. , R. Wang , C. Xie , et al. 2019. “PestNet: An End‐to‐End Deep Learning Approach for Large‐Scale Multiclass Pest Detection and Classification.” IEEE Access 7: 45301–45312.

[fsn371174-bib-0013] Liu, L. , C. Xie , R. Wang , et al. 2020. “Deep Learning Based Automatic Multiclass Wild Pest Monitoring Approach Using Hybrid Global and Local Activated Features.” IEEE Transactions on Industrial Informatics 17, no. 11: 7589–7598.

[fsn371174-bib-0014] Liu, W. , G. Wu , F. Ren , and X. Kang . 2020. “DFF‐ResNet: An Insect Pest Recognition Model Based on Residual Networks.” Big Data Mining and Analytics 3, no. 4: 300–310.

[fsn371174-bib-0015] Malek, M. A. , S. S. Reya , M. Z. Hasan , and S. Hossain . 2021. “A Crop Pest Classification Model Using Deep Learning Techniques.” In In 2021 2nd International Conference on Robotics, Electrical and Signal Processing Techniques (ICREST), 367–371. IEEE.

[fsn371174-bib-0016] Mallick, M. T. , S. Biswas , A. K. Das , H. N. Saha , A. Chakrabarti , and N. Deb . 2023. “Deep Learning‐Based Automated Disease Detection and Pest Classification in Indian Mung Bean.” Multimedia Tools and Applications 82, no. 8: 12017–12041.

[fsn371174-bib-0017] Nanni, L. , G. Maguolo , and F. Pancino . 2020. “Insect Pest Image Detection and Recognition Based on Bio‐Inspired Methods.” Ecological Informatics 57: 101089.

[fsn371174-bib-0018] Nieuwenhuizen, A. T. , J. Hemming , and H. K. Suh . 2018. “Detection and Classification of Insects on Stick‐Traps in a Tomato Crop Using Faster R‐CNN.”

[fsn371174-bib-0019] Pacal, I. , and G. Işık . 2025. “Utilizing Convolutional Neural Networks and Vision Transformers for Precise Corn Leaf Disease Identification.” Neural Computing and Applications 37: 2479–2496. 10.1007/s00521-024-10769-z.

[fsn371174-bib-0020] Pacal, I. , I. Kunduracioglu , M. H. Alma , et al. 2024. “A Systematic Review of Deep Learning Techniques for Plant Diseases.” Artificial Intelligence Review 57: 304. 10.1007/s10462-024-10944-7.

[fsn371174-bib-0021] Pacal, I. 2024. “Enhancing Crop Productivity and Sustainability Through Disease Identification in Maize Leaves: Exploiting a Large Dataset With an Advanced Vision Transformer Model.” Expert Systems With Applications 238: 122099. 10.1016/j.eswa.2023.122099.

[fsn371174-bib-0022] Rani, A. A. , K. L. Prasanna , M. S. Ashraf , A. K. Dey , M. A. A. Walid , and D. R. K. Saikanth . 2023. “Classification for Crop Pest on U‐SegNet.” In 2023 7th International Conference on Computing Methodologies and Communication (ICCMC), 926–932. IEEE.

[fsn371174-bib-0023] Rimal, K. , K. B. Shah , and A. K. Jha . 2023. “Advanced Multiclass Deep Learning Convolution Neural Network Approach for Insect Pest Classification Using TensorFlow.” International Journal of Environmental Science and Technology 20, no. 4: 4003–4016.

[fsn371174-bib-0025] Thenmozhi, K. , and U. S. Reddy . 2019. “Crop Pest Classification Based on Deep Convolutional Neural Network and Transfer Learning.” Computers and Electronics in Agriculture 164: 104906.

[fsn371174-bib-0026] Ullah, N. , J. A. Khan , L. A. Alharbi , A. Raza , W. Khan , and I. Ahmad . 2022. “An Efficient Approach for Crops Pests Recognition and Classification Based on Novel DeepPestNet Deep Learning Model.” IEEE Access 10: 73019–73032.

[fsn371174-bib-0027] Wei, D. , J. Chen , T. Luo , T. Long , and H. Wang . 2022. “Classification of Crop Pests Based on Multi‐Scale Feature Fusion.” Computers and Electronics in Agriculture 194: 106736.

[fsn371174-bib-0028] Xie, C. , R. Wang , J. Zhang , et al. 2018. “Multi‐Level Learning Features for Automatic Classification of Field Crop Pests.” Computers and Electronics in Agriculture 152: 233–241.

